# Caries risk assessment in school children using a reduced Cariogram model without saliva tests

**DOI:** 10.1186/1472-6831-10-5

**Published:** 2010-04-19

**Authors:** Gunnel Hänsel Petersson, Per-Erik Isberg, Svante Twetman

**Affiliations:** 1 Department of Cariology, Faculty of Odontology, Malmö University, Malmö, Sweden; 2 Department of Statistics, Lund University School of Economics and Management, Lund University, Lund, Sweden; 3 Department of Cariology and Endodontics, Faculty of Health Sciences, University of Copenhagen, Denmark

## Abstract

**Background:**

To investigate the caries predictive ability of a reduced Cariogram model without salivary tests in schoolchildren.

**Methods:**

The study group consisted of 392 school children, 10-11 years of age, who volunteered after informed consent. A caries risk assessment was made at baseline with aid of the computer-based Cariogram model and expressed as "the chance of avoiding caries" and the children were divided into five risk groups. The caries increment (ΔDMFS) was extracted from the dental records and bitewing radiographs after 2 years. The reduced Cariogram was processed by omitting the variables "salivary mutans streptococci", "secretion rate" and "buffer capacity" one by one and finally all three. Differences between the total and reduced models were expressed as area under the ROC-curve.

**Results:**

The baseline caries prevalence in the study population was 40% (mean DMFS 0.87 ± 1.35) and the mean 2-year caries increment was 0.51 ± 1.06. Both Cariogram models displayed a statistically relationship with caries development (p < 0.05); more caries was found among those assessed with high risk compared to those with low risk. The combined sensitivity and specificity decreased after exclusion of the salivary tests and a statistically significant reduction of the area under the ROC-curve was displayed compared with the total Cariogram (p < 0.05). Among the salivary variables, omission of the mutans streptococci enumeration impaired the predictive ability the most.

**Conclusions:**

The accuracy of caries prediction in school children was significantly impaired when the Cariogram model was applied without enumeration of salivary tests.

## Background

Risk assessment is an essential component in the decision-making process for the prevention and management of dental caries. Along with the dramatic decline in caries prevalence during the past 30 years [1], the search for acceptable, accurate, and cost-effective strategies for identifying high risk individuals has been intensified and multiple risk factors and indicators have been proposed as targets. A systematic review of literature from the Swedish Council on Technology Assessment in Health Care [2] has however recently concluded that the current methods have a low accuracy, whereas it is more reliable to identify those with a low risk of developing caries. The findings demonstrated, in harmony with several previous reviews [3-5], that there is good evidence to support that past caries experience is the single best predictor for future caries development. This must however be regarded as unsatisfactory since past caries is a "risk factor" that cannot be modified by the therapist and secondly, the goal is to determine the individual caries risk before cavities occur. Thus, there is a need for further development of accurate prediction models.

To facilitate the practical application of caries risk assessment, a computer-based model, the Cariogram [6], has been developed and the predictive ability has been evaluated in three prospective studies in various age groups with a somewhat mixed, but acceptable, outcome [7-9]. In two of the studies with school children and elderly, the Cariogram appeared to predict the increment in a statistically significant way [7,8]. Cariogram is a software program which aims to demonstrate the multi-factorial background of dental caries by illustrating the interaction of nine caries-related factors. Patients are scored on diet, plaque, caries experience, bacterial counts and saliva secretion and the results are shown as a pie-chart risk profile. One possible barrier for the use of this program is the inclusion of salivary tests with microbiological cultivations, such as mutans streptococci enumeration. Chair-side microbial tests are costly and time consuming which delay the process from a patient-motivating point of view. An apparently logical question was therefore whether or not a reduced and "instant" Cariogram, without supplemental laboratory tests, could be applied for caries prediction. The aim of this study was therefore to investigate if a reduced Cariogram model could predict future caries as good as the complete risk assessment model in a group of school children. The null hypothesis was that no differences in the accuracy would be displayed between the two models.

## Methods

### Subjects

This study was performed through a re-evaluation of data previously presented by Hänsel Petersson et al. [7] in which the study group was described in detail. In brief, the baseline study population consisted of 438 school children, 10-11 years of age, who volunteered after informed consent given by the parents. The 2-year follow-up examination comprised of 392 children (89.5%), with a dropout of 46 participants - 23 had moved from the area, 10 were sick or absent at the second examination, 10 did not want to participate in the follow-up study and 3 had not visited the dental clinic during these two years. All the participants were residents of communities with low natural fluoride content (≈0.1 ppm) in the drinking water and reported that they used fluoride toothpaste at least once daily. The original project was approved by the Ethical Committee at Lund University, Sweden.

### Study design and caries scores

The study had a prospective design and the clinical procedure included a questionnaire, an interview, an estimation of oral hygiene and saliva sampling [7]. The samplings and the clinical inspections were carried out by a specially trained nurse and the records and radiographs were scored by two calibrated, experienced dentists and all examinations were blinded. The children were reassessed with the same criteria after 2 years. The regular dental team and the patient were not informed on the risk status during the study. Data on caries experience (DMFS) were extracted from the dental records including bitewing radiographs by the principal investigator and the actual caries increment (ΔDMFS) for each child during the two-year period was calculated. Caries was defined as a marked radiolucency with broken enamel-dentin border or with obvious progression into the dentin. A tooth with fissure sealant was recorded as sound. A re-examination of 29 dental records with radiographs revealed an intra-examiner agreement of 0.96 (Cohen's Kappa).

### Saliva sampling

Paraffin-stimulated whole saliva was collected for 5 minutes for estimation of secretion rate. Salivary mutans streptococci and buffer capacity was determined with Dentocult^® ^SM - Strip mutans and Dentobuff^® ^Strip, respectively. All chair-side tests were obtained from Orion Diagnostica, Espoo, Finland and handled according to the instructions of the manufacturer.

### Risk assessment using the Cariogram

The total Cariogram was created by nine variables entered into the computer program according to Bratthall and Hänsel Petersson [6]. The risk for future caries was expressed as the "percentage chance of avoiding caries in the near future" and the children were divided into five risk groups. In the present study however, more traditional predictive values such as sensitivity, specificity and ROC curves were applied in order to facilitate comparisons between the models. The reduced Cariogram was processed by extracting the risk factors obtained from the saliva sampling ("mutans streptococci counts", "secretion rate" and "buffer capacity") one by one and finally all three. In the reduced model, the factor "diet, content of fermentable carbohydrates", was scored in four levels (from very low intake to poor diet) based on the baseline interviews. The children and their parents were not informed about the outcome of the Cariogram during the study period.

### Statistical methods

All data were processed with the SPSS software (version 17.0, Chicago Ill., USA). For the selected cut off-point, predictive values were calculated with Omnistat, Trelleborg, Sweden. For comparisons between the complete and reduced Cariogram models, the area under the ROC-curve (AUC) was computed and the differences were tested according to Hanley and McNeil [10]. P-values less than 0.05 were considered as statistically significant.

## Results

The caries prevalence in the study population at baseline was 40% (mean DMFS 0.87 ± 1.35) and after two years, 31% of the children had developed new lesions. The mean caries increment (ΔDMFS) was 0.51 ± 1.06. The actual caries incidence (ΔDMFS>0) over two years in the five risk groups assessed with the total and reduced Cariogram is shown in Table 1. Both models displayed a statistically relationship with caries development (p < 0.05); more caries was found among those assessed with high risk compared to those with low risk. Almost all children (99%) remained in the same risk group when the buffer and secretion rate values were aborted. The corresponding value for mutans streptococci elimination was 68% indicating that almost one third of the children changed their risk group, for better or for worse, without use of the salivary mutans streptococci enumeration. The vast majority (74%) were placed in a lower risk category.

**Table 1:** The actual 2-year caries incidence (ΔDMFS>0), expressed as percent, in the five risk groups of school children assessed with the total Cariogram and the reduced model without saliva tests.

	**Percentage chance of avoiding caries**				
	
	**0-20%** **"high risk"**	**21-40%**	**41-60%**	**61-80%**	**81-100%** **"low risk"**
	
**Total Cariogram**	91.7	65.4	58.2	27.2	16.8
**Reduced Cariogram**	100.0	55.3	42.6	18.4	17.9

The table is derived from [7].

**Figure 1:**
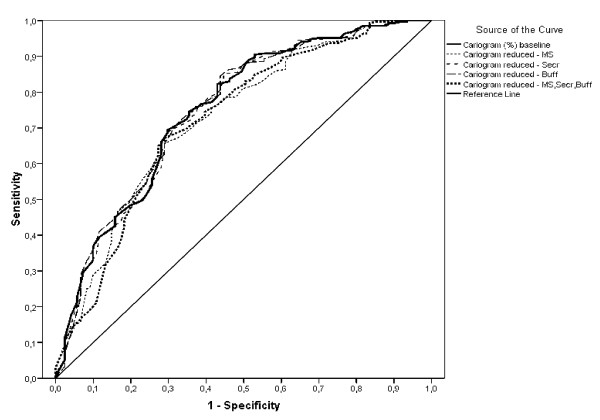
**ROC-curve for the total and the reduced Cariogram**.

The predictive values for an 81-100% chance of avoiding caries (low caries risk) assessed by the total and reduced Cariogram are presented in Table 2. In comparison with the total model, the omission of the salivary parameters increased the sensitivity on expense of a decreased specificity. The combined sensitivity and specificity dropped from 1.33 with the total Cariogram to 1.10 without the salivary tests. This was further displayed in the ROC-curve shown in Figure 1 and in the calculated area under the ROC-curve as presented in Table 3. The total Cariogram was only slightly compromised by the non-use of the variables "buffer capacity" and "secretion rate" while the exclusion of the "mutans streptococci counts" reduced the AUC close to statistical significance (p = 0.055). However, when all the salivary tests were discarded in the reduced model, a statistically significant difference in the area under the ROC curve was displayed (p < 0.05).

**Table 2:** Predictive values (95% CI) for caries increment (ΔDMFS) at the cut-off point "81-100% chance of avoiding caries" in a group of 392 school children assessed by total and reduced Cariogram.

Predictive values	Total Cariogram	Reduced Cariogram			
		**no MS**	**no buffer**	**no secretion rate**	**no MS, no buffer, no secretion rate**
		
**Sensitivity**	0.73 (0.65-0.81)	0.84 (0.77-0.90)	0.79 (0.72-0.86)	0.77 (0.70-0.85)	0.90 (0.85-0.95)
**Specificity**	0.60 (0.54-0.66)	0.47 (0.41-0.52)	0.51 (0.45-0.57)	0.49 (0.44-0.55)	0.20 (0.15-0.25)
**PPV**	0.45 (0.38-0.52)	0.41 (0.35-0.47)	0.42 (0.35-0.48)	0.41 (0.34-0.47)	0.34 (0.28-0.39)
**NPV**	0.83 (0.78-0.88)	0.86 (0.81-0.92)	0.85 (0.79-0.90)	0.83 (0.78-0.89)	0.82 (0.73-0.91)
**PLR**	1.80 (1.50-2.2)	1.60 (1.40-1.80)	1.60 (1.40-1.90)	1.50 (1.30-1.80)	1.10 (1.00-1.20)
**NLR**	0.45 (0.33-0.62)	0.36 (0.23-0.54)	0.41 (0.28-0.59)	0.45 (0.32-0.64)	0.50 (0.28-0.90)

PPV = positive predictive value; NPV = negative predictive value;

PLR = positive likelihood ratio; NLR = negative likelihood ratio

**Table 3:** The mean area under the ROC-curve calculated for total and reduced Cariogram models.

Risk model	AUC^#^	SE^#^	95% CI^#^
**Total Cariogram**	0.751	0.027	0.697-0.804
			
**Reduced Cariogram**			
**no MS**	0.727	0.028	0.672-0.782
**no buffer capacity**	0.751	0.028	0.693-0.802
**no secretion rate**	0.748	0.027	0.698-0.805
**no MS, no buffer, no secretion rate**	0.723*	0.029	0.667-0.780

# area under the ROC-curve, SE = standard error; CI = confidence interval

* significantly different from total Cariogram (p < 0.05; Hanley-McNeil test)

The positive predictive value was fairly low with the total Cariogram and further decreased with the reduced model while the negative predictive value remained virtually the same, around 0.85 (Table 2). Similarly, the positive likelihood ratio decreased from 1.8 with the total Cariogram to 1.1 after application of the reduced model. However, the negative likelihood ratio was found to be less or equal to 0.5 for all the tested models.

## Discussion

Caries risk assessment is one of the cornerstones in patient-centered caries management in order to assist the clinician in the decision-making process concerning treatment, recall appointments and need for additional diagnostic procedures [11]. Apart from having high precision and accuracy, the ideal risk assessment model should be easy to use in the daily practice and utilize inexpensive risk factors that can be scored in a reliable way. In addition, the process should be rapid and the outcome understandable so it can be used as didactic tool in patient motivation. This means that predictive tool should be sensitive enough to catch as many as possible of those with a true caries risk but also correctly identify those with low risk. The Cariogram model is truly comprehensive and illustrates the relative importance of various background factors in an individual risk profile but the increased costs and timely handling of salivary test may have limited its use. The present study was therefore performed to answer the question whether or not the Cariogram model could be of clinical value without the saliva tests. The straight-forward answer based on the positive and negative likelihood ratio was "yes" - it can still be used for caries prediction in school children, and especially to identify those with low risk, but the predictive ability was significantly impaired by exclusion of the saliva tests. Thus, the null hypothesis was rejected. The mutans streptococci counts had the greatest impact on the predictive ability, close to statistical significance (p = 0.055), while the variables "salivary secretion rate" and "buffer capacity" only displayed a small impact on the accuracy. The observation that the mutans streptococci count was a powerful factor in the model was somewhat expected in the light of previous findings [12,13]. In a systematic review from the Swedish Council on Technology Assessment in Health Care [2] it was concluded that the presence of mutans streptococci as a sole predictor for caries development in toddlers during the following 2-3 years had low accuracy. On the other hand, there are a number of studies showing that presence of mutans streptococci, both in plaque or saliva of young caries-free children, appears to be associated with a considerable increase in caries risk [12,13].

It is however important to stress a number of circumstances that should be considered. At first, the findings may be valid only for this age group with a mainly uncompromised saliva secretion rate and buffer capacity; the situation would likely be quite different in an elderly and fragile population with a higher prevalence of hypo-salivation. Secondly, the average caries prevalence and increment was rather low in the study population and only manifest lesions were considered, which may affect the predictive ability and clinical value of any test. Thirdly, the exclusion of a risk value in one of the boxes in the Cariogram model is not the same as "zero". In fact, the computer program estimates a hypothetical value based on a weighed formula based on the rest of the computed variables. In all, seven variables are required to form the risk profile and it is important to emphasize that the different risk groups of the reduced Cariogram exhibited a significant relationship to caries increment. This implies that the reduced model still may be of some value to predict future caries and definitively better than nothing.

The value and accuracy of prediction models must be determined in longitudinal studies and, unfortunately, most papers on risk factors and risk indicators so far describe cross-sectional designs [2]. The fact that there is evidence that past caries experience is the single best predictor for future caries development seems to have been adopted by most clinicians, who apparently more or less ignore the multi-factorial models [14,15]. It is however important to state that weak evidence, or even lack of evidence, does not mean that the variety of risk factors and indicators that are available for the clinician's consideration should be abandoned. The reason for insufficient evidence is most often lack of studies of good quality. For example, plaque amount and tooth morphology may very well be risk factors, although not yet established in an adequate prospective way. Furthermore, the "gut-feeling" among dental professionals, which is almost impossible to define, is a factor that should not be underestimated as shown in the North Carolina risk study [16]. We are well aware of that the Cariogram model has its shortcomings but at this point, we argue that it is more important to carry out a risk assessment incorporating best available evidence, than not to attempt due to lack of firm evidence. Although the combined sensitivity and specificity of the Cariogram model was found to be moderate and average in the present population, the interactive possibility is an important feature in patient motivation.

## Conclusion

In conclusion, the accuracy of caries prediction in school children was significantly impaired when the Cariogram model was applied without enumeration of salivary tests. The mutans streptococci enumeration seemed to be most important of the salivary variables.

## Competing interests

The authors declare that they have no competing interests.

## Authors' contributions

GHP and ST participated in conception and design of the study and GHP supervised and evaluated the data collection. Statistical analyses and interpretation of the results were performed by PI. GHP wrote the manuscript together with ST, PI read the manuscript and all authors approved the final manuscript.

## Pre-publication history

The pre-publication history for this paper can be accessed here:

http://www.biomedcentral.com/1472-6831/10/5/prepub
